# Microbial Consortium Fermentation Remodels the Metabolite Profile and Enhances the Biological Functionality of *Stevia rebaudiana* Leaves

**DOI:** 10.3390/foods15030574

**Published:** 2026-02-05

**Authors:** Guangpeng Chu, Tiejun Chen, Baowei Wang, Shijie Fan, Chaojiang Chen, Yang Deng, Qianru Chen, Jing Wang

**Affiliations:** 1 College of Food Science and Engineering, Qingdao Agricultural University, Qingdao 266109, China; 2 Beijing Huadu Yukou Poultry Industry Co., Ltd., Beijing 101206, China; 3 Feed Research Institute of Chinese Academy of Agricultural Sciences, Beijing 132011, China

**Keywords:** *Stevia rebaudiana*, biotransformation, secondary metabolite remodeling, phenolic acid enrichment, composite fermentation system, redox homeostasis

## Abstract

Microbial fermentation is an effective strategy to enhance the functional value of plant-derived ingredients. In this study, *Stevia rebaudiana* leaves were subjected to microbial fermentation to improve their antioxidant potential and functional properties. A composite fermentation system composed of *Bacillus subtilis* and *Candida utilis* was established through strain screening, and fermentation conditions were optimized using single-factor and orthogonal experiments, with chlorogenic acid (CA) content and antioxidant activity as evaluation indices. The optimal conditions were determined to be a fermentation temperature of 34 °C, a duration of 36 h, a microbial ratio (Bs:Cu) of 2:1, a moisture content of 55%, and an inoculum level of 3%. Under these optimal conditions, fermentation significantly increased CA content, total phenolic and flavonoid levels, and antioxidant capacity compared with unfermented material. Untargeted metabolomic analysis revealed extensive fermentation-induced remodeling of secondary metabolites, particularly phenolic acids, flavonoids, and terpenoids, including the generation of multiple newly formed bioactive compounds. Functional validation using a laying hen model demonstrated that fermented *S. rebaudiana* exhibited enhanced antioxidant and anti-inflammatory status and favorable modulation of physiological indicators compared with unfermented samples. Overall, this study demonstrates that microbial consortium fermentation effectively transforms *S. rebaudiana* from a sweetener-oriented plant into a multifunctional, fermentation-derived functional ingredient. This research is significant as it provides a dual-purpose strategy for developing antioxidant-enriched functional foods for humans and health-promoting natural feed additives for the livestock industry.

## 1. Introduction

Functional foods and health-promoting ingredients have attracted increasing attention due to their potential roles in mitigating oxidative stress, modulating metabolic homeostasis, and supporting overall health [1,2,3]. Recent advances in food science emphasize the development of plant-derived functional ingredients enriched in bioactive compounds, particularly polyphenols, flavonoids, and phenolic acids, which are widely associated with antioxidant and anti-inflammatory activities [4,5]. Among these properties, antioxidant capacity is considered a key indicator for evaluating the functional potential of phytochemical-rich food matrices, as oxidative stress is closely linked to metabolic dysfunction and chronic physiological decline [6]. Oxidative stress stems from an imbalance between pro-oxidants and antioxidant defenses. Dietary phytochemicals, particularly phenolic acids and flavonoids, counteract this by neutralizing reactive oxygen species (ROS) and activating endogenous antioxidant signaling pathways, thereby preventing cellular damage [7,8].

*Stevia rebaudiana* is widely used in the food industry as a natural, non-caloric sweetener due to its high content of steviol glycosides [9,10]. Beyond its sweetening properties, *S. rebaudiana* leaves are rich in chlorogenic acid, flavonoids, and diverse phenolic acids, which have been reported to exhibit antioxidant, anti-inflammatory, and metabolic regulatory activities [11,12]. Chlorogenic acid, one of the major phenolic constituents in *S. rebaudiana*, has received particular attention for its roles in regulating lipid metabolism, glucose homeostasis, and redox balance [13,14]. Currently, *S. rebaudiana* research and industry focus mainly on steviol glycosides due to high market demand and mature extraction technologies. However, other bioactive components like phenolic acids and flavonoids are underutilized. These compounds are trapped within complex plant cell wall matrices, which limits their release and biological activity during standard processing [15].

A major challenge in the utilization of plant-derived bioactive compounds lies in their limited bioaccessibility and bioavailability. In plant tissues, polyphenols and flavonoids often exist in bound or conjugated forms associated with cell wall components, which restrict their release and functional expression during digestion [16]. Conventional processing methods, such as drying or direct extraction, are often insufficient to efficiently liberate these bound phenolics or enhance their biological effectiveness [17]. Therefore, developing effective processing strategies to improve the release and functional expression of plant phenolics is of considerable interest in functional food research.

Microbial fermentation is a traditional and increasingly important food processing technology that has been widely employed to enhance the nutritional and functional properties of plant-based foods. During fermentation, microorganisms secrete a broad spectrum of hydrolytic enzymes capable of degrading plant cell wall components, thereby facilitating the release and transformation of bound phenolic compounds and generating new bioactive metabolites [18,19]. Fermented plant foods have been associated with improved antioxidant capacity, enhanced bioavailability of phytochemicals, and diversified metabolic profiles, highlighting fermentation as a promising strategy for functional food development [20].

*Stevia rebaudiana* has also been preliminarily explored as a substrate for microbial fermentation. Previous studies have reported that lactic acid bacterial fermentation of *S. rebaudiana* can enhance its antioxidant capacity and alleviate oxidative stress in biological models [21,22]. In addition, yeast-mediated fermentation has been shown to induce the biotransformation of steviol diterpenoids, resulting in the generation of novel secondary metabolites [23]. However, existing studies are largely limited to single-strain fermentation or targeted functional observations [21,22]. Systematic investigations focusing on microbial consortium fermentation, comprehensive metabolite remodeling, and subsequent biological validation of fermented *S. rebaudiana* remain scarce.

Compared with monoculture fermentation, microbial consortium fermentation offers complementary enzymatic systems and synergistic metabolic interactions, enabling more efficient substrate utilization and broader metabolite diversification [24]. The integration of untargeted metabolomics further provides a powerful tool for comprehensively characterizing fermentation-induced metabolic remodeling and linking chemical transformations with functional outcomes [25]. Nevertheless, the relationships between microbial consortium fermentation, secondary metabolite remodeling, and biological functionality in *S. rebaudiana* have not yet been fully elucidated.

Functional food research increasingly emphasizes the importance of biological validation beyond in vitro antioxidant assays. Animal models characterized by high metabolic activity and sensitivity to oxidative stress are frequently employed to evaluate the physiological relevance of functional plant-derived ingredients [26]. Laying hens during the late production stage represent a sensitive biological model for assessing antioxidant status, inflammatory responses, and physiological modulation, thereby providing valuable insights into the functional performance of fermented plant ingredients.

Therefore, this study aimed to: (i) develop an efficient microbial consortium fermentation strategy to enhance the antioxidant potential of *S. rebaudiana* leaves; (ii) optimize fermentation conditions to maximize chlorogenic acid content and antioxidant activity; (iii) characterize fermentation-induced metabolic remodeling using untargeted metabolomics; and (iv) evaluate the biological functionality of fermented *S. rebaudiana* using a laying hen model focusing on antioxidant and physiological indicators. This work seeks to provide mechanistic insights and practical guidance for upgrading *S. rebaudiana* from a sweetener-oriented plant into a multifunctional, fermentation-derived functional ingredient. The findings hold substantial significance for both the functional food and animal husbandry industries, offering an effective method to liberate bound phytochemicals and enhance the biological efficacy of plant-derived materials used in health-promoting diets and animal feed.

## 2. Materials and Methods

### 2.1. Experimental Materials

Strain sources: *Bacillus subtilis* (Bs, BNCC 134415), *Lactiplantibacillus plantarum* 1 (Lp1, CICC 20265), *Clostridium butyricum* (Cb, CCTCC M 2018760), and *Candida utilis* (Cu, CICC 31430); *L. plantarum* 2 (Lp2, CICC 25125), *Aspergillus niger* (An, CCTCC M 2018761), *Rhizopus oligosporus* 1 (Ro1, CCTCC M 2020844), and *Rhizopus oligosporus* 2 (Ro2, CCTCC M 2020843) were all preserved in the Laboratory of Fermentation Functional Food Research and Development, Qingdao Agricultural University. *S. rebaudiana* was purchased from Shandong Qinglian Chrysanthemum Industry Co., Ltd. (Qingzhou, Shandong, China). Culture medium: Unless otherwise specified, all synthetic media were obtained from Qingdao Haibo Biotechnology Co., Ltd. (Qingdao, Shandong, China).

### 2.2. Microbial Inoculum Preparation

Preserved *Bacillus subtilis*, *L. plantarum*, *Clostridium butyricum*, and *C. utilis* were activated at 37 °C (28 °C for *C. utilis*) for 24 h. Single colonies were inoculated into respective liquid media and cultured at 37 °C, 130 r/min for 24 h, used as seeds when viable count reached 1 × 10^10^ CFU/mL. To ensure the stability of the strain combination and minimize batch-to-batch variation, each strain was cultured independently under standardized conditions (bacteria at 37 °C, fungi at 28 °C) and harvested at the late exponential growth phase to ensure a consistent physiological state with uniform metabolic activity and high viability for all fermentation batches before being mixed. Glycerol-preserved spore suspensions of *Aspergillus niger* and *Rhizopus oligosporus* were spread on PDA agar, incubated at 28 °C for 4–5 days to induce sporulation. After the spores were scraped, they were resuspended in a sterile solution of 0.9% NaCl and a hemocytometer was used to determine the spore concentration.

### 2.3. Screening of Fermentation Strains

*S. rebaudiana* leaves were dried, pulverized, and passed through a 40-mesh standard sieve. A total of 50.0 g of the leaf powder was weighed, and 50 mL of distilled water (corresponding to a moisture content of 50%) was added. The mixture was soaked for 12 h, sterilized at 121 °C for 15 min, and then cooled to room temperature. Under aseptic conditions, the seed cultures were inoculated at a 2% (*v*/*w*) inoculation volume, or spore suspension was inoculated at 1 × 10^4^ spores/g of substrate. The mixture was thoroughly homogenized, and solid-state fermentation was conducted in 500 mL Erlenmeyer flasks sealed with eight layers of gauze under static conditions to maintain a stable environment. After fermentation, the products were dried at 50 °C to a constant weight, ground, and passed through a 40-mesh sieve. The chlorogenic acid content and antioxidant activity of the fermented products were determined to select the optimal bacterial and fungal fermentation strains.

### 2.4. Single-Factor Experiments

Single-factor experiments assessed the effects of fermentation temperature (22, 25, 28, 31, 34 °C), fermentation time (12, 24, 36, 48, 60, 72 h), moisture content (40%, 45%, 50%, 55%, 60%), inoculation volume (1%, 2%, 3%, 4%, 5%) and strain compound ratio (volume ratios of *Bacillus subtilis* (*B. subtilis*) to *Candida utilis* (*C. utilis*) at 1:1, 1:2, 1:3, 2:1, 3:1) on chlorogenic acid (CA) content and antioxidant activity of fermented *Stevia rebaudiana* leaves, with the remaining four factors fixed at 31 °C, 36 h, 50%, 2%, and 1:1 (*v*/*v*), respectively.

### 2.5. Orthogonal Experiments

A five-factor and five-level orthogonal experimental design [L_25_(5^5^)] was employed to determine the optimal fermentation process conditions for *S. rebaudiana* leaves. The design of the orthogonal experiment is presented in Table S1.

Fermentation experiments were subsequently conducted under the optimized conditions obtained from the orthogonal experiments. The chlorogenic acid content and antioxidant activity were compared among three groups: the product obtained under optimized conditions, the product from the original fermentation conditions, and unfermented *S. rebaudiana* leaves. These comparisons were used to verify the optimization results.

### 2.6. Determination of Chlorogenic Acid Content

The content of CA [27], FRAP [28], and ABTS radical scavenging activity [29] was evaluated based on previous research methods.

Take 0.5 g of sample, sonicate with 10 mL distilled water (360 W, 50 °C, 30 min), then centrifuge (4000 rpm, 10 min). Filter the supernatant through a 0.22 micron membrane filter and measure at 325 nm wavelength using an enzyme-linked immunosorbent assay reader. Quantitative analysis was calibrated using a chlorogenic acid standard curve (0–30 μg/mL, R^2^ > 0.99) prepared under identical conditions.

The assay was performed following a modified method. ABTS working solution (OD_734_ = 0.70 ± 0.02) was prepared by reacting 7.4 mmol/L ABTS with 2.6 mmol/L potassium persulfate in the dark for 16 h. Extracts were mixed with the working solution (1:1, *v*/*v*), incubated for 30 min in the dark, and measured at 734 nm, with Vitamin C as a positive control. The scavenging rate was calculated as: *Scavenging rate* (%) = [1 − (A_1_ − A_2_)/A_0_] × 100.

FRAP was determined using a 2,4,6-tri(2-pyridyl)-s-triazine (TPTZ)-based working solution (pH 3.6 acetate buffer, 10 mM TPTZ, and 20 mM FeCl_3_·6H_2_O at 10:1:1 ratio). Samples were mixed with the working solution and water (1:30:3), incubated in the dark for 30 min, and measured at 593 nm. Results were quantified against a FeSO_4_·7H_2_O standard curve (0.1–2.5 mM).

### 2.7. Widely Targeted Metabolomics Analysis

*S. rebaudiana* leaf powder was sterilized and cooled to obtain the unfermented sample SR. After fermentation under optimal conditions, the fermented sample FSR was obtained. The sample was rapidly frozen in liquid nitrogen and stored at −80 °C. The widely targeted secondary metabolites were obtained from Wuhan MetWare Biotechnology Co., Ltd. (Wuhan, Hubei, China).

Metabolite extraction followed the protocol described in the analyst report, using 30 mg of lyophilized powder and 70% methanol containing internal standards. Analysis was performed on a UPLC-MS/MS system (ExionLC AD and Tandem MS, SCIEX, Fremont, CA, USA). Metabolites were identified via the MWDB database based on MS2 spectra, excluding redundant signals. For data quality control, QC samples were analyzed every 10 injections, and features with CV > 0.5 were removed. Multivariate statistical analysis (PCA, OPLS-DA) utilized log transformation and UV scaling to normalize data. Differential metabolites were screened using criteria of VIP > 1 and FC ≥ 2 or ≤0.5.

### 2.8. Animal Experiment Design

A total of 432 66-week-old Jinfen No. 6 laying hens with similar egg production rates (75%) and body weights were selected for the experiment. Prior to the trial, all hens were fed a basal diet. The study lasted 7 weeks, with the first week dedicated to experimental dietary adaptation. All hens were randomly assigned to three treatment groups, each with six replicates of 24 hens per replicate. Group settings are detailed in Table 1. Throughout the study, the photoperiod was set to 16 h light–8 h dark, and all hens were provided ad libitum access to feed and water. Other husbandry practices followed the 2023 guidelines outlined in the Jingfen No. 6 Management Manual.

### 2.9. Egg and Serum Sampling and Analysis

Daily egg production records were maintained for each group, including total eggs laid, broken eggs, egg weight, and total feed intake. The feed conversion ratio per replicate was determined by dividing total feed intake by overall egg weight. Using data collected over the six-week trial period, average daily feed intake, mean egg weight, egg production rate, broken egg rate, and feed-to-egg ratio were computed. In the sixth week, 18 hens (3 per replicate) were randomly chosen for blood collection via the sub-wing vein. Blood samples were stored at −20 °C for subsequent serum analysis.

Serum levels of inflammatory cytokines, antioxidants, sex hormones, and immune markers were measured with corresponding ELISA kits (Shanghai Tongwei Biotechnology Co., Ltd., Shanghai, China) following the manufacturer’s instructions.

### 2.10. Data Statistics and Analysis

All experiments were conducted in triplicate. Statistical analyses were performed using analysis of variance followed by Duncan’s multiple range test to determine significant differences among groups. Data processing and visualization were conducted using Origin 8.5 software. In figures and tables, different lowercase letters indicate statistically significant differences at the *p* < 0.05 level.

## 3. Results and Discussion

### 3.1. Selection of Fermentative Cultures

**Figure 1:**
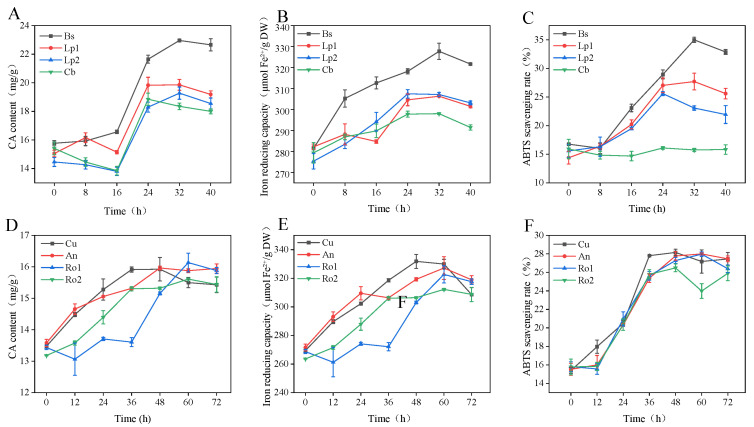
Effects of different fermentation strains on chlorogenic acid content and antioxidant activity of *S. rebaudiana* leaves. (**A**) CA content of different bacteria; (**B**) FRAP values of different bacteria; (**C**) ABTS values of different bacteria; (**D**) CA content of different fungi; (**E**) FRAP values of different fungi; (**F**) ABTS values of different fungi.

As bacterial strains vary in their capacities to utilize nutrients within fermentation substrates, selecting appropriate strains is critical for improving fermentation efficiency [30]. Figure 1A–C present the evaluation metrics for bacterial fermentation, including CA content, ferric ion reduction capacity, and ABTS radical scavenging activity. The CA content exhibited a consistent trend of an initial increase followed by a decline across all strains. Among them, *B. subtilis* achieved the highest CA content (22.73 mg/g at 32 h), corresponding to a 41.03% increase compared with unfermented samples. Its ferric ion reduction power and ABTS radical scavenging rate reached 327.72 μmol Fe^2+^/g dry weight (DW) and 35%, respectively, values significantly higher than those of other bacterial strains. The differences observed among strains primarily result from their distinct metabolic characteristics. Compared with *B. subtilis*, *L. plantarum,* and *C. butyricum* exhibit weaker capabilities in secreting cell wall-degrading enzymes, such as cellulase and xylanase [31,32]. Based on the comprehensive evaluation of the three dynamic indicators shown in Figure 1A–C, *B. subtilis* was selected as the optimal bacterial strain for the fermentation of *S. rebaudiana*.

Figure 1D–F depict the dynamic changes in the three indicators during fungal fermentation. After 36 h of fermentation, *C. utilis* increased the CA content by 17.82% to 15.9 mg/g, significantly exceeding that obtained from other fungal strains (Figure 1D). Its ferric ion-reducing capacity and ABTS radical scavenging rate reached 331.83 μmol Fe^2+^/g DW and 27.8%, respectively. Although *A. niger* and *R. oligosporus* can secrete cellulase and hemicellulase to decompose plant cell walls and facilitate the release of intracellular substances, they also produce oxidases (e.g., polyphenol oxidase and peroxidase). These enzymes directly catalyze the oxidation of flavonoids and polyphenols, leading to structural damage (e.g., oxidation of phenolic hydroxyl groups and disruption of conjugated systems) and subsequent reduction in antioxidant activity [33]. Based on the integrated assessment of the three parameters, *C. utilis* was selected as the optimal fungal fermentation strain.

### 3.2. Effects of Single-Strain and Composite-Strain Fermentation on Chlorogenic Acid and Antioxidant Activity

**Figure 2:**
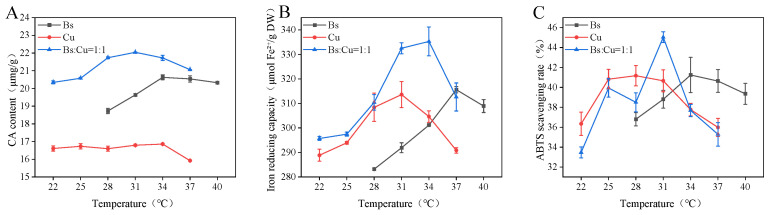
Effects of single and composite strains on fermentation performance. (**A**) CA content; (**B**) FRAP values; (**C**) ABTS values.

Figure 2 illustrates the effects of single- and composite-strain fermentations on CA content and antioxidant activity. Overall, the composite-strain fermentation system demonstrated superior performance compared with single-strain fermentation. Microbial interactions in co-culture systems are inherently complex, and composite inoculations generally exhibit enhanced metabolic efficiency relative to monocultures [34]. As shown in Figure 2A, during 36 h fermentation across different temperatures, the composite-strain system consistently produced higher CA contents than any single-strain fermentation, with the highest CA content (22.05 mg/g) being achieved at 31 °C. Similarly, both the ferric ion-reducing power and the ABTS radical scavenging rate were significantly higher (*p* < 0.05) in the composite fermentation group than in the single-strain groups, reaching maximum values of 335.28 μmol Fe^2+^/g DW (Figure 2B) and 45.02% (Figure 2C), respectively. This enhancement can be attributed to the synergistic interaction between *B. subtilis* and *C. utilis*. *B. subtilis* secretes cellulase and xylanase, which effectively degrade the plant cell wall and promote the release of intracellular bioactive compounds such as CA, flavonoids, and other polyphenols [35]. Throughout this process, pentose metabolites, including xylose, are generated as intermediate products that can be used by *C. utilis* to synthesize further target metabolites. The findings reported by Chen et al. on synergistic fermentation effects are consistent with these results [36]. Therefore, the co-culture of *C. utilis* and *B. subtilis* demonstrates significant synergistic advantages over single-strain systems, representing a promising strategy for improving the fermentation performance and functional value of *S. rebaudiana*.

### 3.3. Optimization of Fermentation Conditions

**Figure 3:**
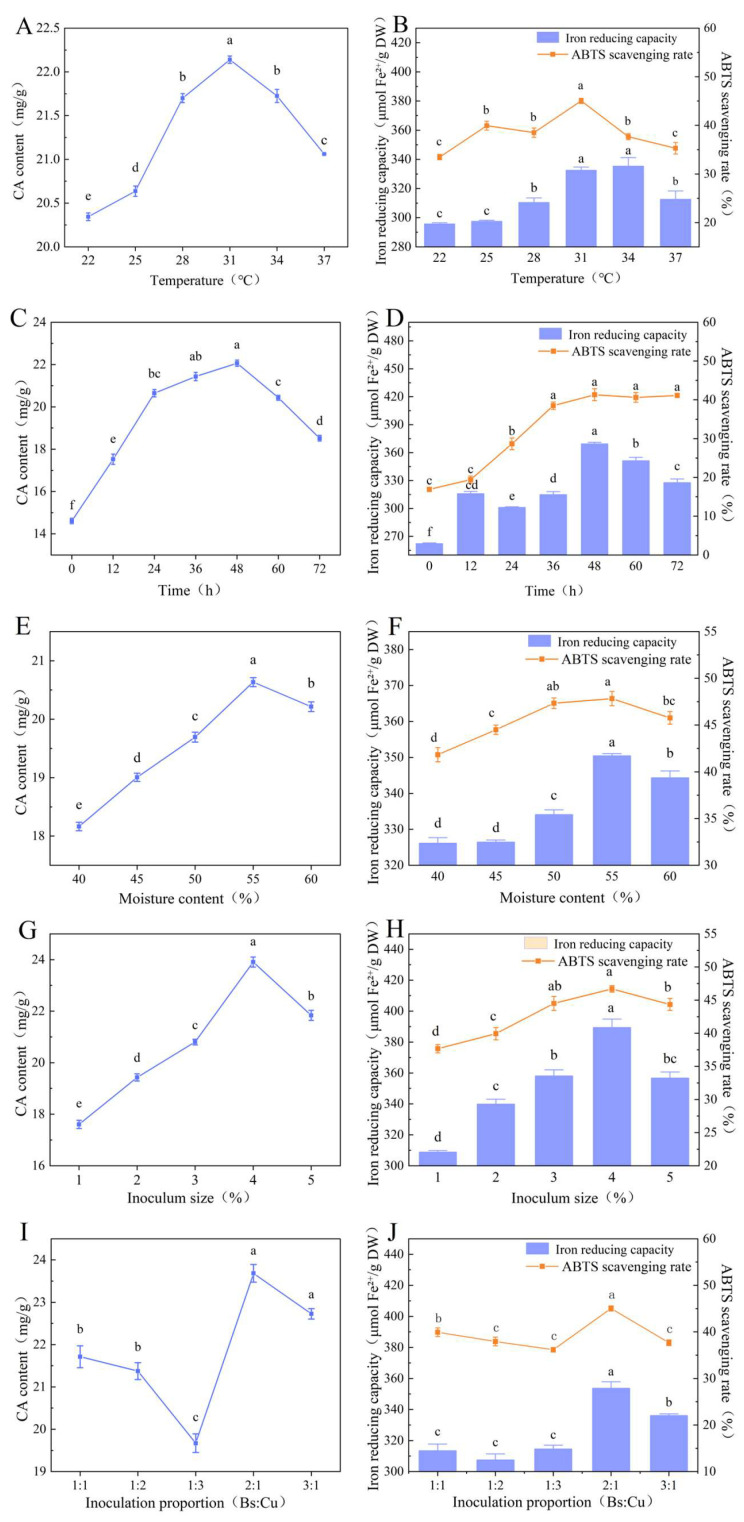
Results of single-factor experiment of *S. rebaudiana* cultivation using *B. subtilis*–*C. utilis* composite fermentation. (**A**) CA content at different temperatures; (**B**) antioxidant values at different temperatures; (**C**) CA content at different times; (**D**) antioxidant values at different times; (**E**) CA content at different substrate water contents; (**F**) antioxidant values at different substrate water contents; (**G**) CA content at different inoculum sizes; (**H**) antioxidant values at different inoculum sizes; (**I**) CA content at different microbial ratios; (**J**) antioxidant values at different microbial ratios. Note: different lowercase letters indicate significant differences between groups (*p* < 0.05).

Fermentation temperature significantly affects the physicochemical environment, enzymatic activity, and metabolic pathways throughout the fermentation process, thereby influencing both reaction kinetics and final product quality [37]. As shown in Figure 3A, CA content gradually increased with rising temperature, peaking at 22.15 mg/g at 31 °C before declining. Similarly, the ferric ion reducing capacity rose with increasing temperature and then decreased beyond the optimal range, with the highest values observed at 31 and 34 °C (*p* > 0.05). The highest ABTS scavenging rate also peaked at 31 °C, reaching 45% (Figure 3B). At lower temperatures, reduced membrane fluidity limits microbial growth, whereas excessive temperatures accelerate microbial senescence and death [38]. Therefore, 31 °C was identified as the optimal fermentation temperature for the composite microbial system.

Fermentation involves microbial proliferation, metabolic activity, and substrate degradation, and the duration of fermentation may influence process efficiency and cost-effectiveness [38]. As shown in Figure 3C, CA content increased significantly with fermentation time, rising from 14.5 mg/g at 0 h to 21.97 mg/g at 48 h (Figure 3C). The ferric ion reducing capacity peaked at 370 μmol Fe^2+^/g DW at 48 h (Figure 3D), while the ABTS scavenging capacity peaked at 36 h and showed no significant change thereafter (Figure 3D). Considering that both CA content and reducing power, key indicators of fermentation efficiency, continued to increase up to 48 h, 48 h was determined to be the optimal fermentation duration for the *B. subtilis-C. utilis* consortium.

Substrate moisture content plays a key role in regulating plant cell wall degradation and the release of bioactive compounds by affecting microbial enzyme activity and substrate mass transfer efficiency [39]. As shown in Figure 3E, CA content first increased and then decreased with rising substrate moisture content, reaching a maximum of 20.6 mg/g at 55% moisture. The antioxidant indices displayed a similar trend (Figure 3F): both ferric ion reducing capacity and ABTS radical scavenging rate peaked at 55% moisture, consistent with the CA content pattern. In solid-state fermentation systems, the regulatory relationship between substrate moisture and microbial metabolism is nonlinear. Instead, an “optimal moisture content range” exists that matches the physiological demands of microorganisms. Within this range, microorganisms maintain vigorous growth and efficient metabolism, whereas deviation markedly inhibits their physiological activity [40]. Therefore, 55% was identified as the optimal moisture content for the composite microbial fermentation system.

Inoculum level directly influences the secretion of extracellular enzymes (e.g., cellulase, xylanase) by modulating microbial population density [41]. As shown in Figure 3G, CA content exhibited a clear increasing trend with higher inoculum levels. When the inoculum level rose from 1% to 4%, CA content increased from 17.5 mg/g to 23.9 mg/g DW. At low inoculum levels, limited enzyme secretion resulted in slower cellulose degradation [42]. Conversely, excessively high inoculum levels accelerated nutrient depletion in the early fermentation phase, altering microbial metabolic balance. The antioxidant parameters followed a pattern consistent with CA content, reaching peak values at a 4% inoculum level, 382 μmol Fe^2+^/g DW for ferric ion reduction capacity, and 46.7% for ABTS scavenging rate (Figure 3H). However, overly dense microbial populations elevate intracellular reactive oxygen species (ROS), promoting rapid antioxidant consumption to maintain redox equilibrium [43]. Accordingly, 4% was determined as the optimal inoculum concentration for the composite fermentation process.

The inoculation ratio of *B. subtilis* to *C. utilis* had a significant impact on the fermentation outcomes (Figure 3I,J). As the proportion of *B. subtilis* increased, both CA content and antioxidant activity first increased and then declined. When the ratio of *B. subtilis* to *C. utilis* increased from 1:1 to 1:3, CA content gradually decreased, accompanied by a similar downward trend in antioxidant capacity. At a 2:1 ratio, the CA content, ferric ion reducing capacity, and ABTS radical scavenging rate reached peak values of 23.67 mg/g, 353.65 μmol Fe^2+^/g DW, and 45.17%, respectively. Further increasing the proportion of *B. subtilis:C. utilis* to 3:1 led to a decline in all three parameters. This trend suggests that, compared with *C. utilis*, *B. subtilis* secretes a wider spectrum of hydrolytic enzymes capable of degrading plant cell walls, thereby promoting the release of free antioxidants such as polyphenols and flavonoids. These findings demonstrate that the inoculation ratio of microbial strains is a key factor influencing synergistic interactions and overall fermentation efficiency. Therefore, an optimal inoculation ratio of *B. subtilis*:*C. utilis* = 2:1 was selected for subsequent experiments.

### 3.4. Orthogonal Experiment

Building upon the single-factor experiments, an L_25_ (5^5^) orthogonal design was employed to optimize the fermentation parameters, using ferric ion reducing capacity as the evaluation index. The experimental results are summarized in Table S2. Based on the R values, the order of factors affecting the ferric ion reduction capacity is A (temperature) > B (fermentation time) > C (microbial ratio) > D (moisture content) > E (inoculum amount). The K value represents the mean value for each level of the main effect. By comparing K_1_, K_2_, K_3_, etc., the optimal level for each factor was determined. According to the K-values, the optimal conditions are A_4_, B_3_, C_4_, D_3_, E_3_, corresponding to a temperature of 34 °C, a fermentation time of 36 h, a microbial ratio of 2:1, a moisture content of 55%, and an inoculum level of 3%.

**Figure 4:**
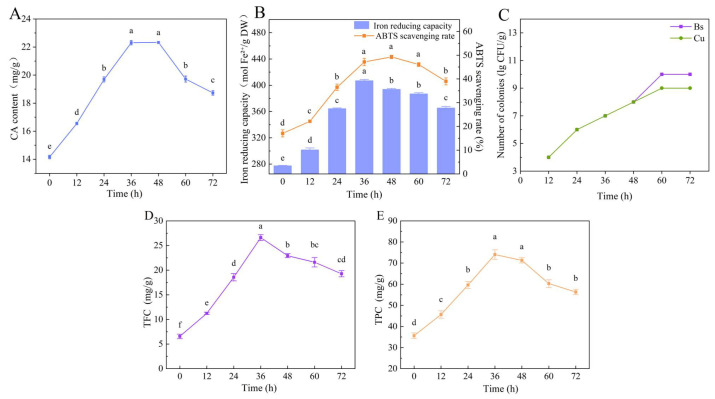
Validation experiment for optimal fermentation process. (**A**) CA content; (**B**) antioxidant capacity; (**C**) colony count; (**D**) TFC content; (**E**) TPC content. Note: different lowercase letters indicate significant differences between groups (*p* < 0.05).

Verification Experiment: The optimal fermentation process derived from the orthogonal test was applied to ferment *S. rebaudiana* for the validation of optimization results, with the simultaneous determination of its antioxidant capacity. (Figure 4). Under these optimized parameters, the ferric ion reduction capacity reached 406 μmol Fe^2+^/g DW, exceeding the maximum value observed in the orthogonal test and confirming the reliability of the established optimal preparation process. Compared with the unfermented control, fermentation under optimal conditions resulted in a 56% increase in CA content, reaching 22.34 mg/g (Figure 4A); a 150% increase in ABTS scavenging capacity (Figure 4B); a 45.6% improvement in ferric ion reduction capacity (Figure 4B); and total viable counts of *B. subtilis* and *C. utilis* both exceeding 10^7^ CFU/g (Figure 4C).

### 3.5. Dynamic Changes in Total Phenolic and Total Flavonoid Content at Different Fermentation Durations

Similarly to most plant tissues, polyphenols and flavonoids in *S. rebaudiana* exist in both bound and free forms [44]. During fermentation, microorganisms secrete hydrolyze enzymes that convert bound phenolic compounds into their free counterparts, thereby enhancing their bioavailability and antioxidant potential [45]. As shown in Figure 4, both total flavonoid content (TFC) and total phenolic content (TPC) in fermented *S. rebaudiana* exhibited a characteristic increase followed by a decrease over the fermentation period, a trend consistent with results reported by Wang et al. [46]. Compared with the unfermented control (0 h), both TFC and TPC significantly increased (*p* < 0.05) during the early and middle stages of fermentation. At 36 h, TFC and TPC reached 26.60 mg/g and 74.04 mg/g, respectively, representing 4.3-fold and 2.08-fold increases relative to the baseline values. The initial increase in TPC and TFC can be attributed to the rapid microbial proliferation and active secretion of enzymes, which degrade plant cell wall components, such as cellulose, proteins, and pectin, and thereby facilitate the release of bound phenolics and flavonoids [47]. In the later stages of fermentation, these released phenolic and flavonoid compounds are partially utilized, degraded, and transformed by microorganisms, leading to a decrease in their overall content [48].

### 3.6. Differential Metabolites

**Figure 5:**
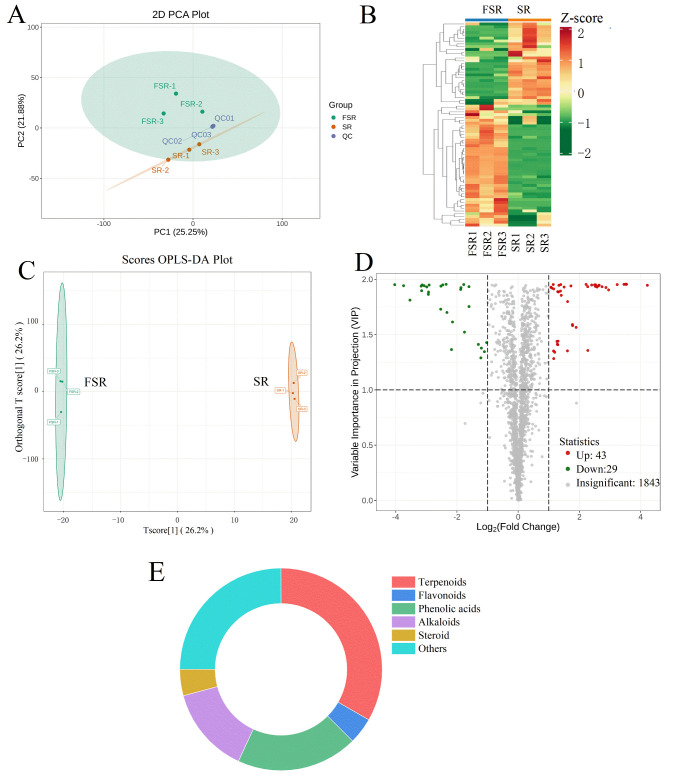
Multivariate analysis of identified metabolites. (**A**) PCA analysis; (**B**) HCA authentication; (**C**) OPLS-DA model diagram; (**D**) Volcano plot of differential metabolites; (**E**) Pie chart of differential metabolites.

Based on the preliminary orthogonal optimization (Section 3.4) and the dynamic accumulation profiles shown in Figure 4, the 36 h time point was selected for downstream analyses. This duration represents the stage where chlorogenic acid (CA) content and overall antioxidant capacity reach their peaks, providing functionally potent material for metabolomic and biological validation. PCA was applied under an unsupervised model to evaluate overall metabolic variation among samples. As shown in Figure 5A, PCA clearly separated unfermented *S. rebaudiana* (SR) and fermented *S. rebaudiana* (FSR) from the quality control (QC, was prepared by mixing sample extracts for evaluating repeatability of samples with identical treatment) samples. The tight clustering of QC data points indicated high analytical reproducibility of both sample preparation and detection processes. Among the first (PC1) and second (PC2) principal components, which together explained 47.13% of the total variance (PC1: 25.25%; PC2: 21.88%), SR and FSR samples were distributed into clearly distinct regions, confirming that fermentation induces significant alterations in *S. rebaudiana*’s metabolite composition. A type of log_10_ transformation was applied to the peak area data prior to HCA to minimize the effect of absolute peak intensity differences on pattern recognition. HCA grouped metabolites with similar expression profiles and revealed clear separation between SR and FSR samples, as illustrated in Figure 5B. The HCA heatmap demonstrated that SR and FSR were separated into two distinct metabolic profiles, indicating that microbial metabolic activity during fermentation profoundly reshaped *S. rebaudiana*’s metabolic landscape. In order to further validate these observations, an orthogonal partial least squares-discriminant analysis (OPLS-DA) model was constructed to distinguish the metabolic profiles of SR and FSR samples. The key model parameters, R^2^X (explained variance of the dataset), R^2^Y (explained variance of the grouping), and Q^2^ (predictive ability of the model), all indicated high model stability and reliability. The score plot of the OPLS-DA model is presented in Figure 5C. Specifically, R^2^Y approached 1 and Q^2^ exceeded 0.5, confirming the robustness and predictive reliability. Based on variable importance in projection (VIP) > 1 and a fold change (FC) ≥ 2 or ≤0.5, a total of 72 differential metabolites (DMs) were identified between SR and FSR (Supplementary Table S3). Among these, 43 metabolites were upregulated and 29 were downregulated following fermentation (Figure 5D). These DMs were classified into 6 major categories: terpenoids (33.3%), flavonoids (4.2%), phenolic acids (19.4%), alkaloids (13.9%), steroids (4.2%), and other compounds (25.0%) (Figure 5E). Correlation analysis further revealed a strong negative correlation between metabolites that increased and those that decreased post-fermentation, while metabolites within the same group (either upregulated or downregulated) exhibited significant positive correlations. This pattern suggests two potential mechanisms: (1) certain metabolites that decrease during fermentation may be converted into newly increased compounds; and (2) microbial enzymatic activity may enhance the release of bound or conjugated active components. Overall, these results confirm that fermentation significantly modulates the abundance of bioactive metabolites in natural plant matrices, likely through a combination of metabolic transformation and enzymatic release mechanisms [49].

### 3.7. Differences in Secondary Metabolites Between SR and FSR

**Figure 6:**
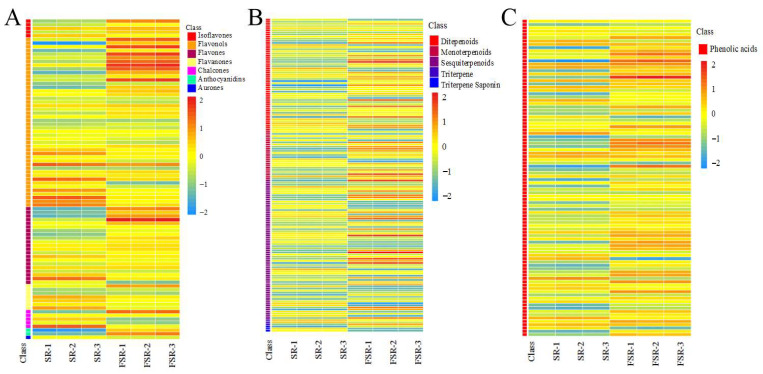
Heatmap of metabolite differences. (**A**) Flavonoids, (**B**) Terpenoids, (**C**) Phenolic acids.

The flavonoids detected in *S. rebaudiana* were classified into flavones, flavanols, isoflavones, chalcones, naringins, dihydroflavones, and anthocyanins. As shown in Figure 6A, fermentation markedly altered both the composition and relative abundance of flavonoids. Differential metabolite enrichment indicated that the altered compounds were primarily involved in flavonoid biosynthesis, isoflavonoid biosynthesis, and flavanol biosynthesis pathways. A total of 24 new flavonoid metabolites were generated post-fermentation (Table S4), including 7 flavones, 14 flavanols, 1 aurone, 2 chalcones, and 2 dihydroflavones (naringenin-7-O-rutinoside-4′-O-glucoside and naringenin). Compared with SR, 68 flavonoid compounds were significantly upregulated in FSR, likely contributing to enhanced antioxidant activity after fermentation. Microbial fermentation can also modify flavonoid structures, thereby improving their bioavailability and biological efficacy [50]. Notably, the newly formed isovitexin-7-O-glucoside in FSR exhibits hepatoprotective effects [51,52], while the newly generated hesperidin displays anticancer properties [53]. These results demonstrate that microbial fermentation can drive the biosynthesis of novel bioactive compounds, providing a promising strategy for enhancing the functional and applicational value of *S. rebaudiana*.

The terpenoids identified in *S. rebaudiana* were classified into diterpenoids, monoterpenoids, sesquiterpenoids, triterpenoids, and triterpenoid saponins. As shown in Figure 6B, fermentation significantly altered the terpenoid profile, primarily in diterpenoids, monoterpenoids, sesquiterpenoids, and triterpenoids. A total of 21 new terpenoid metabolites were detected post-fermentation, including 11 diterpenoids, 9 sesquiterpenoids, and 1 triterpenoid saponin, as shown in Table S5. Among these, the diterpene compound 16-Hydroxyferruginol exhibits strong anti-inflammatory and antioxidant properties [52], while the sesquiterpene compound Jiangxibaiyingsu H synergistically enhances antimicrobial activity with flavonoids by inhibiting bacterial biofilm formation and quorum-sensing pathways [54]. These findings highlight microbial fermentation as an effective strategy for generating pharmacologically valuable terpenoid derivatives from natural substrates.

A total of 108 phenolic acid differential metabolites were identified (Figure 6C). Nine phenolic acids were newly detected in FSR but absent in SR, including isochlorogenic acid A, B, and C; p-coumaroylgalactosylamine; 4-feruloyl-5-caffeoylquinic acid; 3-hydroxybenzoic acid; 2-hydroxycinnamic acid; 4-O-feruloylquinic acid; and ferulic acid. Ferulic acid has been widely reported to possess anti-inflammatory, antioxidant, skin-whitening, and anti-wrinkle activities [55]. Importantly, isochlorogenic acids A, B, and C are newly generated after *S. rebaudiana* fermentation, exhibiting potent antioxidant, anti-inflammatory, and antibacterial activities. These compounds have potential applications in the food, pharmaceutical, and cosmetic fields [56]. Collectively, these findings indicate that microbial fermentation effectively promotes the synthesis and diversification of phenolic acid compounds.

To better understand the biological significance of the fermentation process, the 72 identified differential metabolites were prioritized based on their functional roles (Table S3). Phenolic acids (14 compounds) and terpenoids (24 compounds) were identified as the primary biological drivers of the observed effects. Specifically, the significant enrichment of caffeoyl-glucose derivatives and sesquiterpenoids (e.g., Bigelovin) represents the major biologically relevant change, as these classes are well-recognized for their potent antioxidant and anti-inflammatory properties. In contrast, although 19 compounds categorized as ‘Others’ (including furfurals like 5-HMF and sugar derivatives) showed high fold changes, they were classified as minor fermentation markers resulting from substrate degradation, with secondary impact on the animal’s physiological status. This prioritization clarifies that the core functional benefits of FSR are predominantly attributable to the targeted remodeling of the phenolic and terpenoid profiles.

The metabolic remodeling observed in this study represents a significant advancement over existing *S. rebaudiana* fermentation strategies. Previous research has demonstrated that fermentation with lactic acid bacteria (e.g., *L. plantarum*) can improve the antioxidant potential of *Stevia* and alleviate oxidative stress in biological models [21,22]. However, these studies typically focus on the release of common phenolics or the modification of steviol glycosides to improve specific clinical symptoms like alcohol poisoning [21,22]. Similarly, single-strain yeast fermentations have been reported to target the biotransformation of specific diterpenoids into novel derivatives [23]. In contrast, our microbial consortium approach induced a more comprehensive global remodeling of the metabolite profile, as evidenced by the identification of 72 differential metabolites across six major chemical classes. Notably, the synergistic action of *B. subtilis* and *C. utilis* led to the generation of 24 new flavonoids and 21 new terpenoids that were not reported in monoculture systems. This broad-spectrum transformation provides a mechanistic basis for the superior biological functionality observed in the laying hen model compared to unfermented controls.

### 3.8. Laying Performance and Egg Quality

To evaluate the biological functionality of fermented *S. rebaudiana* as a health-promoting ingredient, its effects on egg quality, nutritional characteristics, and related physiological outcomes were further examined using a laying hen model. As expected, (Table S6), compared with SR, FSR supplementation resulted in improved egg quality traits and supporting production-related parameters. Compared to the SR group, the FSR group demonstrated significantly higher egg production rate, total egg production, average egg weight, and proportion of conforming eggs. Concurrently, the FSR group exhibited a significantly reduced feed-to-egg ratio, along with markedly lower rates of broken eggs, feces-contaminated eggs, and bruised eggs. Research indicates that the feed-to-egg ratio of 80-week-old laying hens fed fermented feed decreased, consistent with the findings of this study [57].

### 3.9. Serum Marker Analysis

**Figure 7:**
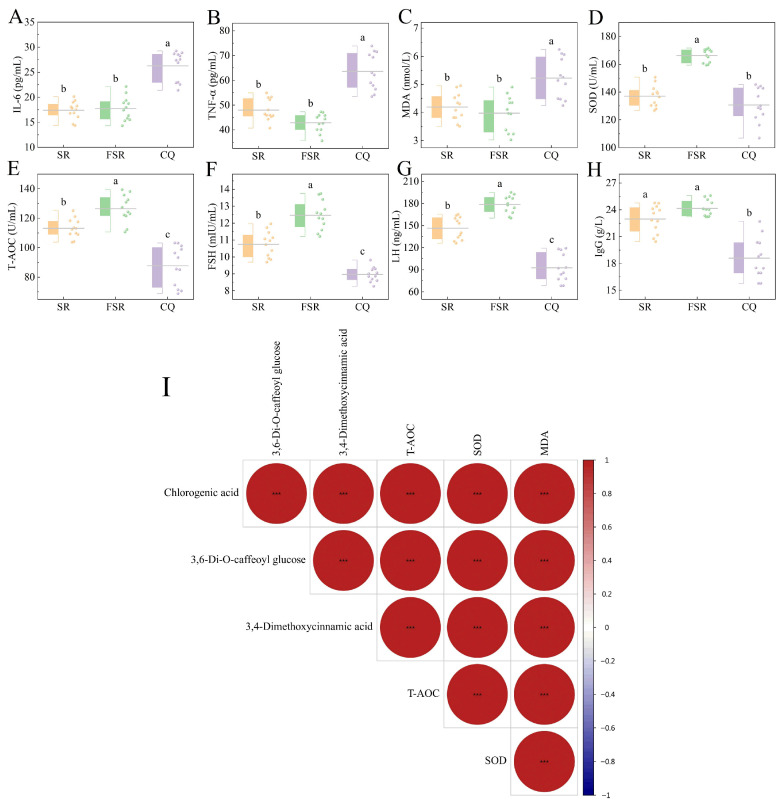
Effects of fermented *S. rebaudiana* on serum indicators in late-laying hens and their correlation with key metabolites. (**A**) IL-6, (**B**) TNF-α, (**C**) MDA, (**D**) SOD, (**E**) T-AOC, (**F**) FSH, (**G**) LF, (**H**) IgG, (**I**) Pearson correlation analysis. Different lowercase letters (a, b, c) indicate significant differences among groups at *p* < 0.05, and “***” denotes extremely significant differences at *p* < 0.001.

Microbial fermentation significantly enhanced the biological functionality of *S. rebaudiana*, as evidenced by improved antioxidant status, inflammatory markers, and endocrine-related indicators. Compared with the CQ (Figure 7), both the SR and FSR groups significantly reduced levels of the inflammatory factors IL-6 and TNF-α, as well as the oxidative damage marker MDA. Meanwhile, they significantly increased antioxidant capacity (T-AOC), serum gonadotropin levels (FSH and LH) and immunoglobulin G (IgG) levels. The FSR group demonstrated significantly higher levels of serum SOD, T-AOC, FSH and LH than the SR group. This is attributed to fermentation-induced degradation of antinutritional factors, synthesis of novel bioactive metabolites, and enhanced bioavailability of natural functional components. Previous studies have demonstrated that microbial fermentation enhances the bioavailability and biological efficacy of plant polyphenols, thereby modulating oxidative stress and inflammatory responses in vivo [58,59]. The elevated endocrine-related indicators observed in the FSR may further reflect improved redox homeostasis and functional signaling mediated by fermentation-derived bioactive metabolites.

To further elucidate the intrinsic relationship between fermentation-induced metabolite remodeling and biological efficacy, Pearson correlation analysis was performed between high-priority metabolites (chlorogenic acid, 3,6-Di-O-caffeoyl glucose, and 2,3-Di-O-caffeoyl glucose) and antioxidant indicators (SOD, T-AOC, and MDA) (Figure 7I). The results indicated that the three key phenolic acids exhibited highly significant positive correlations with serum SOD activity and T-AOC levels (R^2^ > 0.9, *p* < 0.01), which strongly demonstrated that the phenolic acid compounds produced during fermentation serve as the direct material basis for enhancing the antioxidant status of the body. Furthermore, although MDA levels in this experiment showed a positive correlation fluctuation alongside the increase in metabolites, the synergistic enhancement of the overall antioxidant system significantly improved the physiological status of the laying hens. This data-driven correlation analysis provides solid statistical support for the study’s conclusion that microbial transformation enhances functional activities.

### 3.10. Industrial Perspectives and Challenges

From the perspective of biosafety and industrial application, the *B. subtilis* and *C. utilis* strains used in this study are allowed to be added to the feed and meet the current feed additive standards and requirements. From an economic perspective, the FSR evaluated in this study has significant biological benefits, including a reduction in the feed–egg ratio and the improvement of production performance, which has economically feasible potential. As a functional feed additive, FSR has broad application prospects in the field of animal husbandry. Large-scale industrial solid-state fermentation often faces common problems such as uneven mass and heat transfer, fluctuation of substrate moisture content, bacterial contamination, and difficulties in process monitoring. The stability of the fermentation process and the conversion rate of the product can be effectively improved by adopting the technical strategies of forced ventilation and segmented temperature control, precise spray control of humidity, sterile closed inoculation, and step-by-step amplification of small-scale, pilot-scale, and large-scale experiments.

## 4. Conclusions

Microbial consortium fermentation effectively enhanced the functional potential of *Stevia rebaudiana* by increasing chlorogenic acid content, phenolic and flavonoid levels, and antioxidant capacity. Untargeted metabolomics revealed extensive fermentation-induced remodeling of secondary metabolites, particularly phenolic acids, flavonoids, and terpenoids. Functional validation using a laying hen model demonstrated improved antioxidant and anti-inflammatory status, supporting a close link between metabolic transformation and biological functionality. Overall, microbial fermentation represents a promising approach to upgrading *S. rebaudiana* from a sweetener-oriented plant into a multifunctional, fermentation-derived functional ingredient. Future research should focus on formal toxicological evaluations and cross-species trials to assess the long-term cumulative effects of FSR on animal health and product quality.

## Figures and Tables

**Table 1:** Feeding Different Diet Compositions.

Group	Diet Composition
SR Group	Base diet + 1% *S. rebaudiana*
FSR Group	Base diet + 1% fermented *S. rebaudiana*
CQ Group	Base diet

## Data Availability

Data supporting this study are included within the manuscript or Supplementary Information files.

## Supplementary Materials

The following supporting information can be downloaded at: https://www.mdpi.com/article/10.3390/foods15030574/s1, Table S1: Design of Orthogonal Experiment; Table S2: Results of Orthogonal Test Design; Table S3: Significant Differential Metabolites; Table S4: 24 New Flavonoid Compounds Produced by Fermentation; Table S5: 21 New Terpenoid Compounds Produced by Fermentation; Table S6: Effects of Fermented *S. rebaudiana* on Laying Performance in Laying Hens.
