# Supplementary material for: Microbial Consortium Fermentation Remodels the Metabolite Profile and Enhances the Biological Functionality of Stevia rebaudiana Leaves
Source: Foods. 2026 Feb 5;15(3):574. doi: 10.3390/foods15030574 (PMC12896879; doi:10.3390/foods15030574)
Supplement: Supplementary file 1 [file foods-15-00574-s001.zip › foods-4110592-supplementary.pdf]

Microbial Consortium Fermentation Remodels the Metabolite Profile and Enhances the Biological Functionality of *Stevia rebaudiana* Leaves

Guangpeng Chu [1], Tiejun Chen [1,*], Baowei Wang [1], Shijie Fan [2], Chaojiang Chen [2], Yang Deng [1], Qianru Chen [1] and Jing Wang [3]

1 College of Food Science and Engineering, Qingdao Agricultural University, Qingdao 266109, China

2 Beijing Huadu Yukou Poultry Industry Co., Ltd., Beijing 101206, China

3 Feed Research Institute of Chinese Academy of Agricultural Sciences, Beijing 132011, China

* Correspondence: chentiejun@qau.edu.cn; Tel./Fax: +86-532-58957973

Table S1 Design of Orthogonal Experiment

No.	Temperature （A）(℃)	Time （B）(h)	Moisture Content （C）(%)	Inoculation Voiume （D）(%)	Strain Ratio （E）
1	22	12	40	1	1：1
2	22	24	50	4	3：1
3	22	36	60	2	2：1
4	22	48	45	5	1：3
5	22	60	55	3	1：2
6	25	12	60	4	1：3
7	25	24	45	2	1：2
8	25	36	55	5	1：1
9	25	48	40	3	3：1
10	25	60	50	1	2：1
11	28	12	55	2	3：1
12	28	24	40	5	2：1
13	28	36	50	3	1：3
14	28	48	60	1	1：2
15	28	60	45	4	1：1
16	31	12	50	5	1：2
17	31	24	60	3	1：1
18	31	36	45	1	3：1
19	31	48	55	4	2：1
20	31	60	40	2	1：3
21	34	12	45	3	2：1
22	34	24	55	1	1：3
23	34	36	40	4	1：2
24	34	48	50	2	1：1
25	34	60	60	5	3：1

Table S2 Results of Orthogonal Test Design

No	Temperature (A) (℃)	Time (B) (h)	Moisture Content (C) (%)	Inoculation Voiume (D) (%)	Temperature (A) (℃)	Iron reduction capacity (Mmol Fe^{2+}/g DW)
1	1	1	1	1	1	335.12
2	1	2	3	4	5	338.46
3	1	3	5	2	4	345.58
4	1	4	2	5	3	352.93
5	1	5	4	3	2	344.01
6	2	1	5	4	3	343.71
7	2	2	2	2	2	345.02
8	2	3	4	5	1	359.74
9	2	4	1	3	5	360.64
10	2	5	3	1	4	363.56
11	3	1	4	2	5	349.58
12	3	2	1	5	4	377.23
13	3	3	3	3	3	386.96
14	3	4	5	1	2	374.02
15	3	5	2	4	1	372.15
16	4	1	3	5	2	360.23
17	4	2	5	3	1	369.02
18	4	3	2	1	5	371.68
19	4	4	4	4	4	392.19
20	4	5	1	2	3	372.14
21	5	1	2	3	4	366.36
22	5	2	4	1	3	362.08
23	5	3	1	4	2	394.67
24	5	4	3	2	1	351.70
25	5	5	5	5	5	357.40
K1	343.22	351.00	362.54	363.95	357.12	
K2	354.53	358.37	352.46	352.24	352.05	
K3	371.99	371.72	366.03	364.45	352.74	
K4	373.05	366.30	353.77	363.18	366.09	
K5	366.44	361.85	365.47	356.74	357.11	
R	29.83	20.723	13.584	12.21	14.04	

Table S3 Significant Differential Metabolites

**Compounds**	**Class**	VIP	P-value	Fold_Change	Type
5,8a-dimethyl-1,2,3,4,5,6,7,8-octahydronaphthalene-1,4a,7-triol	Terpenoids	1.89	0.02	2.49	up
Botryosphaerinone	Terpenoids	1.95	0.01	0.13	down
Dictamnol	Terpenoids	1.92	0.01	2.14	up
Balsamiferine E	Terpenoids	1.91	0.00	2.23	up
Liguhodgsonal	Terpenoids	1.94	0.00	0.17	down
1,6-O,O-Diacetylbritannilactone	Terpenoids	1.93	0.01	2.83	up
JiangxiBaiyingsu H	Terpenoids	1.95	0.00	4.65	up
Eujavanoic acid B	Terpenoids	1.41	0.25	2.42	up
7-Oxo-pimara-8,11,15-trien-2-ol	Terpenoids	1.95	0.01	0.18	down
11,12,13-tris-nor-trans-Eudesm-5-en-7-one	Terpenoids	1.95	0.01	0.11	down
7-Hydroxy-1-{4-Hydroxy-2,2,6-Trimethyl-7-Oxabicyclo[4.1.0]Heptan-1-Yl}-3-Methylocta-3,5-Dien-2-one	Terpenoids	1.95	0.01	9.40	up
Geijeron	Terpenoids	1.70	0.01	0.20	down
Samboginone	Terpenoids	1.44	0.12	2.40	up
Apiosporic acid	Terpenoids	1.95	0.00	0.20	down
Dehydrolinalool	Terpenoids	1.94	0.03	0.07	down
Bigelovin	Terpenoids	1.90	0.08	7.74	up
Procurcumenol	Terpenoids	1.58	0.03	3.43	up
Grayanotoxin VII	Terpenoids	1.95	0.01	5.64	up
12-O-methylspruceanone	Terpenoids	1.35	0.20	0.47	down
3-Methoxytanapartholide	Terpenoids	1.59	0.03	3.42	up

Leoheteronin C	Terpenoids	1.36	0.21	0.22	down
2,6,10,14-tetramethylhexadeca-2,6,10,14-tetraene-1,4,16-triol	Terpenoids	1.35	0.18	2.23	up
Viscidic Acid A	Terpenoids	1.93	0.03	7.28	up
Acasenterpene B*	Terpenoids	1.57	0.06	3.71	up
3-[6-O-(4-Hydroxy-trans-cinnamoyl)-beta-D-glucopyranosyloxy]-3-methylglutaric acid	Phenolic acids	1.29	0.21	0.43	down
Phenyllactate	Phenolic acids	1.75	0.03	0.33	down
Methyl 4-hydroxybenzoate	Phenolic acids	1.93	0.00	4.83	up
3,4-Dimethoxycinnamic acid	Phenolic acids	1.93	0.01	2.10	up
3,6-Di-O-caffeoyl glucose*	Phenolic acids	1.93	0.02	6.67	up
2,3-Di-O-caffeoyl glucose*	Phenolic acids	1.94	0.02	5.75	up
1,4-O-di-Caffeoyl-3-O-glucoside Quinic Acid*	Phenolic acids	1.44	0.12	2.47	up
Phenylacetic acid	Phenolic acids	1.34	0.21	2.29	up
4-Hydroxybenzoyl acetyl glucoside	Phenolic acids	1.41	0.15	2.46	up
Dehydrocinnzeylanol	Phenolic acids	1.90	0.06	0.11	down
Tropate	Phenolic acids	1.93	0.03	0.13	down
p-Coumaroylferuloyltartaric acid	Phenolic acids	1.28	0.23	2.24	up
1-O-p-coumaroyl Dihydroxyvaleric acid	Phenolic acids	1.61	0.07	0.23	down
Picein	Phenolic acids	1.35	0.14	3.05	up
Okanin-3',4'-di-O-glucoside*	Flavonoids	1.43	0.13	0.49	down
Naringenin-7-O-Rutinoside-4'-O-glucoside	Flavonoids	1.94	0.02	6.16	up
Phellatin	Flavonoids	1.38	0.17	0.43	down
(2S)-2-amino-2-methylpentanoic acid	Alkaloids	1.95	0.00	2.62	up
1-Methyl-2-[(Z)-6-undecyl]-4(1H)-quinolone	Alkaloids	1.92	0.00	0.27	down
Piperidine	Alkaloids	1.95	0.00	2.21	up
Verpacamide A	Alkaloids	1.91	0.04	0.27	down

Isatin	Alkaloids	1.89	0.00	2.61	up
Retronecine	Alkaloids	1.94	0.01	3.30	down
O-Acetylcarnitine	Alkaloids	1.95	0.00	0.29	down
N-[(4-methoxyphenyl)methyl]-2-(7H-purin-6-ylsulfanyl)acetamide	Alkaloids	1.81	0.01	0.09	down
N1-Methyl-4-pyridone-5-carboxamide	Alkaloids	1.95	0.00	0.06	down
Allantoin	Alkaloids	1.73	0.01	0.17	down
5,8-Epidioxyergosta-6,22-dien-3-ol (Ergosterol peroxide)	Steroids	1.52	0.09	0.30	down
Wortmannine D	Steroids	1.88	0.09	0.13	down
9alpha-hydroxy-androst-4-ene-3,17-dione	Steroids	1.95	0.01	5.78	up
6-phenyl-hexan-2-ol	Others	1.86	0.00	2.65	up
3-butyl-5-methyl-5h-furan-2-one	Others	1.80	0.08	3.06	up
Ieodomycin B	Others	1.94	0.02	0.12	down
Phloroglucinol	Others	1.95	0.00	10.95	up
5-Hydroxymethyl-2-furaldehyde*	Others	1.96	0.00	11.31	up
5-hydroxymethylenefural*	Others	1.96	0.00	11.60	up
5-Methoxyfurfural*	Others	1.96	0.00	11.57	up
3-Oxo-Alpha-Ionol,4-(3-Hydroxy-1-Butenyl)-3,5,5-Trimethyl-2-Cyclohexen-1-One	Others	1.36	0.22	4.84	up
4-Hydroxy-β-bulnesene	Others	1.41	0.12	0.41	down
6-Tuliposide A*	Others	1.86	0.12	0.13	down
Soraphen O	Others	1.89	0.00	2.45	up
4-Hydroxybenzaldehyde	Others	1.95	0.00	18.61	up
4-Hydroxy-3-methoxy-benzaldehyde	Others	1.95	0.00	5.28	up
(5S,8R,12S)-5,8-dihydroxy-12-methyl-1-oxacyclododeca-3,6-dien-2-one	Others	1.95	0.00	6.25	up

2-Methyl-d-erythritol 2,4-cyclodiphosphate(2,4,7-trihydroxy-6-(hydroxymethyl)-6-methyl-1,3,5,2,4-trioxadiphosphocane 2,4-dioxide)	Others	1.95	0.00	2.52	up
Methyl Alpha-D-Galactopyranoside*	Others	1.93	0.00	0.33	down
7-deoxy-sedoheptulose*	Others	1.93	0.03	5.96	up
8-[3-(1-Hydroxyoct-2-EN-1-YL)oxiran-2-YL]octanoic acid	Others	1.94	0.03	0.11	down

Table S4 24 New Flavonoid Compounds Produced by Fermentation

Flavonoids	**Compounds**
	6-Hydroxyluteolin 7-xylosyl-(1->6)-glucoside*
	Resokaempherol
	isovitexin-7-O-glucoside
	6-Hydroxyluteolin 5-glucoside*
	6-Hydroxyluteolin 7-sambubioside*
	Nepetin-7-O-glucoside(Nepitrin)*
Flavones	6-Hydroxyluteolin 7-xylosyl-(1->6)-glucoside*
	Kaempferol-3-O-galactoside*
	Multinoside A*
	6-Hydroxykaempferol-6,7-O-diglucoside
	Kaempferol-3-O-(apiofuranosyl-(1'''-2''))-galactopyranoside*
Flavonols	3-{[(2R,3S,4R,5S,6S)-6-({[(2S,3R,4S)-3,4-dihydroxy-4-(hydroxymethyl)oxolan-2-yl]oxy}methyl)-3,4,5-trihydroxyoxan-2-yl]oxy}-5,7-dihydroxy-2-(4-hydroxyphenyl)chromen-4-one*
	Herbacetin 3-glucoside*
	Quercetin 7-O-sambubioside*
	Quercitrin
	Quercetin-3,7-Di-O-glucoside*
	Meratin*
	Quercetin-3-O-(2'''-coumaroyl)glucosyl-(1→2)-glucoside
	Kaempferol-3-O-galactoside*
	Multinoside A*
	6-Hydroxykaempferol-6,7-O-diglucoside
Aurones	Aureusidin 4,6-diglucoside*
Chalcones	4,2',4',6'-Tetrahydroxy-3-methoxychalcone
	4,2',3',4'-Tetrahydroxychalcone*
Dihydroflavones	Naringenin-7-O-Rutinoside-4'-O-glucoside
	Naringenin*

Table S5 21 New Terpenoid Compounds Produced by Fermentation

Terpenoids	**Compounds**
Diterpenoids	7-Hydroxy-1-{4-Hydroxy-2,2,6-Trimethyl-7-Oxabicyclo[4.1.0]Heptan-1-Yl}-3-Methylocta-3,5-Dien-2-one
	Viscidic Acid A
	(14S)-14,20-Cyclolabda-8,13(16)-dien-19-ol
	2,4b,8-trimethyl-3,4,4a,5,6,7,8a,9-octahydro-1H-phenanthrene-1,8-diol
	16-Hydroxyferruginol*
	Vitexilactone C
	19-Nor-3,5-Clerodadien-15-Oic Acid
	[5-Methyl-2-(6-Methylhepta-1,5-Dien-2-Yl)-9-Methylidenecyclonon-5-En-1-Yl]Methyl Acetate*
	1-ethyl-11a-methyl-1H,2H,3H,3aH,3bH,4H,6H,7H,8H,9H,9aH,9bH,10H,11H,11aH-cyclopenta[a]phenanthren-1-ol*
	Norflickinflimiod F
	4-[3-(4,8-Dimethyl-3,7-Nonadienyl)-3-Methyloxiranyl]-2-Butanone
Hemiterpenoids	JiangxiBaiyingsu H
	1,1,7,7a-tetramethyl-1a,2,3,5,6,7,7a,7b-octahydro-1H-cyclopropa[a]naphthalene*
	Balsamiferine E
	1(10),8-Aristoladiene
	11-Hydroxy-13-Isopropyl-4(15),10(14)-Guaiadien-12,6-olide
	Dendronobilin I*
	Maali-1,3-Diene
	eupatorinol
	3-Methoxytanapartholide
Triterpenoid Saponins	Medicagenic acid 3-O-triglucoside

Table S6 Effects of Fermented *S. rebaudiana* on Laying Performance in Laying Hens

**Item**	SR	FSR	CQ	SEM	P
1-3W					
Feed-to-Egg Ratio	2.61±0.16	2.65±0.19	2.71±0.15	0.036	0.074
Manure-stained Egg Rate	9.79±1.53a	9.16±1.82b	10.19±1.50a	0.329	0.037*
Broken Egg Rate	1.59±0.73	1.53±0.79	1.59±0.60	0.155	0.926
Bruised Egg Rate	2.19±0.86b	2.30±1.36b	2.99±1.00a	0.245	0.012*
Total Egg Number	106.14±5.05	108.32±6.21	105.77±6.41	1.239	0.198
Average Egg Weight	53.61±1.39	53.45±2.32	53.52±1.54	0.394	0.96
45-52g Egg Rate	26.99±5.57	29.54±5.95	28.21±3.82	1.065	0.172
52-60g Egg Rate	44.10±7.28a	41.52±4.54b	40.07±4.22b	1.046	0.031*
60-65g Egg Rate	4.82±1.75	5.05±1.44	5.24±1.63	0.309	0.533
Qualified Egg Rate	75.92±6.39	76.11±6.66	73.53±5.88	1.224	0.244
Laying Rate	73.71±3.50	75.22±4.31	73.45±4.45	0.819	0.176
3-6W					
Feed-to-Egg Ratio	2.55±0.14b	2.45±0.10c	2.72±0.09a	0.024	<0.001**
Manure-stained Egg Rate	6.94±1.67b	5.87±1.18c	8.64±1.11a	0.354	<0.001**
Broken Egg Rate	0.86±0.87b	0.64±0.64b	1.96±1.03a	0.187	<0.001**
Bruised Egg Rate	2.49±1.45	1.59±1.20	2.67±1.57	0.318	0.061
Total Egg Number	104.71±5.33b	110.48±4.24a	102.14±7.08b	1.104	0.001**
Average Egg Weight	54.64±1.14ab	55.00±1.19a	53.42±1.44b	0.284	0.002**
45-52g Egg Rate	21.63±2.84	22.70±3.12	20.73±3.53	0.646	0.146
52-60g Egg Rate	48.48±6.20ab	49.37±4.61a	44.95±4.78b	1.062	0.027*
60-65g Egg Rate	6.76±1.99	7.72±2.50	5.97±2.48	0.519	0.053
Qualified Egg Rate	76.86±6.42b	79.79±4.64a	71.66±4.71c	1.056	<0.001**
Laying Rate	72.72±3.70b	76.72±2.94a	70.93±4.92b	0.846	<0.001**
